# Targeted Proteomic Quantitation of NRF2 Signaling and Predictive Biomarkers in HNSCC

**DOI:** 10.1016/j.mcpro.2023.100647

**Published:** 2023-09-15

**Authors:** Nathan T. Wamsley, Emily M. Wilkerson, Li Guan, Kyle M. LaPak, Travis P. Schrank, Brittany J. Holmes, Robert W. Sprung, Petra Erdmann Gilmore, Sophie P. Gerndt, Ryan S. Jackson, Randal C. Paniello, Patrik Pipkorn, Sidharth V. Puram, Jason T. Rich, Reid R. Townsend, José P. Zevallos, Paul Zolkind, Quynh-Thu Le, Dennis Goldfarb, Michael B. Major

**Affiliations:** 1 Department of Cell Biology and Physiology, Washington University in St Louis, St Louis, Missouri, USA; 2 Department of Radiation Oncology, Stanford University, Stanford, California, USA; 3 Lineberger Comprehensive Cancer Center, University of North Carolina at Chapel Hill, Chapel Hill, North Carolina, USA; 4 Department of Pathology, Stanford University School of Medicine, Stanford, California, USA; 5 Department of Medicine, Washington University School of Medicine, St Louis, Missouri, USA; 6 Department of Otolaryngology/Head and Neck Surgery, Washington University School of Medicine, St Louis, Missouri, USA; 7 Institute for Informatics, Washington University in St Louis, St Louis, Missouri, USA

**Keywords:** PRM, IS-PRM, SureQuant, targeted proteomics, HNSCC, FFPE, NRF2, HPV

## Abstract

The NFE2L2 (NRF2) oncogene and transcription factor drives a gene expression program that promotes cancer progression, metabolic reprogramming, immune evasion, and chemoradiation resistance. Patient stratification by NRF2 activity may guide treatment decisions to improve outcome. Here, we developed a mass spectrometry–based targeted proteomics assay based on internal standard–triggered parallel reaction monitoring to quantify 69 NRF2 pathway components and targets, as well as 21 proteins of broad clinical significance in head and neck squamous cell carcinoma (HNSCC). We improved an existing internal standard–triggered parallel reaction monitoring acquisition algorithm, called SureQuant, to increase throughput, sensitivity, and precision. Testing the optimized platform on 27 lung and upper aerodigestive cancer cell models revealed 35 NRF2 responsive proteins. In formalin-fixed paraffin-embedded HNSCCs, NRF2 signaling intensity positively correlated with NRF2-activating mutations and with SOX2 protein expression. Protein markers of T-cell infiltration correlated positively with one another and with human papilloma virus infection status. CDKN2A (p16) protein expression positively correlated with the human papilloma virus oncogenic E7 protein and confirmed the presence of translationally active virus. This work establishes a clinically actionable HNSCC protein biomarker assay capable of quantifying over 600 peptides from frozen or formalin-fixed paraffin-embedded archived tissues in under 90 min.

## Highlights

• Optimized data acquisition reduces cycle times for triggered PRM experiments.
• PRM robustly quantifies NRF2 activation in cell lines and FFPE oral cavity tumors.
• Detection of low-abundant immune checkpoints and HPV16 E7 from oropharyngeal tumors.
• HPV(+) oropharyngeal cancers overexpress T-cell markers relative to HPV(−) cancers.

## Graphical Abstract

**Figure:**
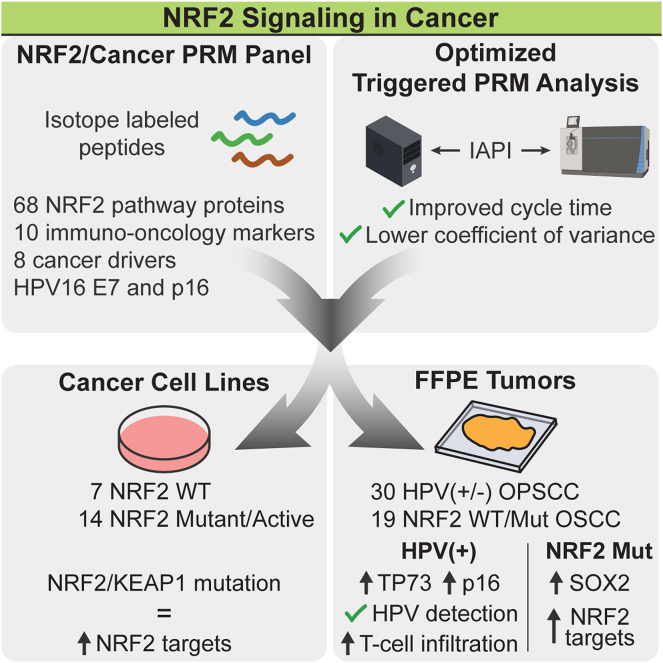

## In Brief

Optimized internal standard–triggered parallel reaction monitoring (OIS-PRM) reduces cycle times and increases sensitivity. OIS-PRM was applied with a custom peptide library to study protein markers of NRF2 signaling, HPV infection, and tumor-infiltrating immune cells. NRF2 activity was robustly quantified in cell lines and HNSCC FFPE tumors. Moreover, OIS-PRM detected HPV16 E7 and revealed increased T-cell marker and immune checkpoint proteins in HPV(+) compared to HPV(−) tumors.

Head and neck squamous cell carcinoma (HNSCC) is the seventh most common cancer worldwide; in the United States, 66,000 new cases and 15,000 deaths were expected in 2022 (1, 2). Key risk factors include alcohol consumption, tobacco use, and human papilloma virus (HPV) infection (1). Immunohistochemistry (IHC) staining for CDKN2A (p16) serves as a proxy for HPV status and is the leading prognostic biomarker in oropharyngeal squamous cell carcinoma (3). HPV(+) tumors of the oropharynx are associated with a more favorable prognosis with a 75 to 80% 5-years survival rate than HPV(−) HNSCC tumors which portend a 45 to 50% 5-years survival rate (3, 4). For locoregionally advanced disease, chemoradiation or surgery with radiation (RT) ± chemotherapy has remained the standard of care treatment for decades, with no meaningful improvement in overall survival (1, 5). Most recently, the addition of immune checkpoint inhibitors (ICI) to the therapeutic armament for recurrent and metastatic HNSCC has improved outcomes as ICI elicits durable responses in just under 20% of these patients (6, 7).

Molecular characterization efforts that identify treatment responsive, nonresponsive, and recurrent HNSCC have revealed several key determinants of patient outcome and have potential to maximize the effective use of these therapeutic options. Specifically in HPV(−) HNSCCs, constitutive activation of the NRF2 oxidative stress response pathway prognosticates poor overall survival and predicts locoregional failure following RT (8, 9, 10, 11, 12, 13, 14). Mutations that drive constitutive NRF2 activation occur in 17% of these cancers (15). Despite the predictive power and high frequency of NRF2 activation in cancer, clinical assays that leverage NRF2 signaling to stratify patients for improved therapeutic response remain to be developed and proven. Another predictive biomarker for HNSCC therapy, PD-L1 cumulative positivity score, has been used in the clinic to predict ICI responsiveness but only achieves a receiver operating characteristic of 0.62 in recurrent/metastatic HNSCC (16). A T-cell–inflamed gene expression profile has demonstrated better predictive power than PD-L1 CPS, yet it still leaves room for improvement (16, 17, 18, 19). Given this current landscape of predictive biomarkers for use in HNSCC, we developed a targeted proteomics assay that quantifies markers for the following: (1) NRF2 signal transduction, (2) HNSCC tumor suppressors and oncogenes, (3) immuno-oncology signaling, and (4) HPV infection.

Constitutive NRF2 signaling drives RT resistance and locoregional failure in HPV(−) HNSCC. In normal cells, the KEAP1/CUL3 E3 ubiquitin ligase complex binds NRF2 and catalyzes its ubiquitylation for subsequent proteasomal degradation (20, 21, 22). Metabolic, oxidative, and electrophilic stressors inhibit NRF2 degradation by KEAP1/CUL3, resulting in NRF2 stabilization, nuclear translocation, and transcriptional activation of target genes that collectively restore cell health (23). These targets encode enzymes supporting antioxidant pathways, drug metabolizing enzymes, components of the pentose phosphate pathway, and others (15, 23). We recently showed that NRF2-activating mutations predict locoregional failure in locally advanced oral and larynx cancers (13, 14). However, prior mutation-based studies fail to account for many NRF2 active cancers that lack a known mutational driver (15). A robust, fast, and cost-effective NRF2 activity diagnostic assay has potential to guide patient treatment decisions, including radiation dose or treatment modality (24, 25).

Effective biomarkers might also predict successful immune checkpoint inhibition, a therapy to which fewer than one-in-five HNSCC patients respond (17, 18, 26). Two widely studied prognostic indicators are PD-L1 expression and T-cell–inflamed gene expression profile (GEP) (17, 19). However, IHC staining for PD-L1 fails to reliably predict ICI response in the majority of HNSCC patients (16, 18, 27). GEP predicts outcomes more reliably with much lower false-positivity rates (16, 18), but both methods have limitations. Immunostaining assays are confounded by poor correspondence of IHC scores to molar abundance, covalent protein modifications, and functional redundancy (*e.g.* PD-L2), and GEP considers mRNAs that may correlate poorly with their protein counterparts (28, 29, 30, 31). Lastly, a growing body of evidence suggests that an active NRF2 pathway reduces the strength of antitumor immunity (14, 18, 32, 33, 34, 35, 36). In the context of an inflamed tumor microenvironment, NRF2 promotes PD-L1 expression, the recruitment of immunosuppressive myeloid cells, and M2 macrophage polarization (14, 18, 32, 33, 34). A mass spectrometry (MS)-based proteomics tool to quantify both NRF2 signaling and the presence and functions of leukocytes in the tumor microenvironment might improve prediction of therapeutic response and empower future studies of an NRF2-immune infiltration axis in cancer (18, 32, 33, 34, 35, 36).

This work presents an optimized proteomics assay for the study of biomarkers for NRF2 signaling proteins, HNSCC-associated cancer drivers, T-cell infiltration, and HPV infection. The technology is based on a custom implementation of internal standard–triggered parallel reaction monitoring (IS-PRM), which leverages stable isotope labeled (SIL) peptides to direct efficient data acquisition (37). IS-PRM enables relative and absolute quantitation of many hundreds of analytes at low attomolar abundance from minimal sample input, which is suitable to quantify many transcription factors, kinases, and other scarce signaling molecules from tumor biopsies and archival tissue blocks (37, 38, 39, 40). We benchmarked our optimized IS-PRM (OIS-PRM) method against a commercial implementation called SureQuant to establish its improved performance (38, 41). We applied OIS-PRM to study NRF2 signaling components and targets in genetically engineered cell models and a cohort of genotyped lung, esophageal, and head and neck cancer cell lines. Additionally, in two patient cohorts of formalin-fixed paraffin embedded (FFPE) HNSCC tumors, we quantified protein expression for tumor-immune infiltration, pan-squamous cell carcinoma cancer drivers, HPV infection, and NRF2-related proteins.

## Experimental Procedures

### Cell Culture and Lentiviral Transduction

All cell lines were maintained in a humidified incubator at 37 °C with 5% CO2. Cell line identities were validated by short tandem repeat analysis (LabCorp, Genetica Cell Line Testing), and cultures were regularly tested for *mycoplasma* contamination (Lonza). The UPCI:SCC090 (CRL-3239), UPCI:SCC152 (CRL-3240), and UPCI:SCC154 (CRL-3241) cell lines were purchased from ATCC and cultured in Eagle's Minimum Essential Media (Corning) supplemented with 10% fetal bovine serum (Sigma), 1% penicillin–streptomycin (Corning), and 2 mmol/L ʟ-glutamine (GIBCO). HEK293T cells (CRL-11268) were purchased from ATCC and cultured in DMEM (Corning) supplemented with 10% fetal bovine serum and 1% penicillin–streptomycin.

Recombinant lentivirus was produced in HEK293T cells using PEI-based transfection. Briefly, psPAX2 packaging (Addgene #12260), VSV-G envelope (Addgene #12259), and UBC-driven NRF2 E79Q vectors were combined with PEI at a 3:1 ratio (μl PEI: μg DNA). Supernatants containing virus were filtered and added to SCC90, SCC152, and SCC154 cells. Transduced cells were selected with 50 μg/ml, 50 μg/ml, and 250 μg/ml hygromycin, respectively.

### Immunoblotting

Cell lines were grown to 70 to 80% confluence and lysed in RIPA (10% glycerol, 50 mM Tris–HCl pH 7.4, 150 mM NaCl, 2 mM EDTA, 0.1% SDS, 1% NP40, 0.2% sodium deoxycholate, aqueous) containing protease and phosphatase inhibitors (Thermo Fisher Scientific) and benzonase (Santa Cruz). Protein concentrations were determined using the bicinchoninic acid (BCA) Protein Assay Kit, equally loaded and separated by SDS-PAGE, transferred to a nitrocellulose membrane, blocked in 5% milk, and incubated with primary antibodies overnight at 4 °C. Washed membranes were incubated for 45 min at room temperature in secondary antibody solution (LI-COR IRDye 680, 800; 1:10,000 in 5% milk), imaged on an Odyssey CLx, and analyzed using Image Studio Software (https://www.licor.com/bio/image-studio/). Antibodies were used at the following dilutions: β-actin (MilliporeSigma #A5316, 1:5000), HMOX1 (Abcam #ab13243, 1:1000), NRF2 (Cell Signaling Technology #20733, 1:1000), and NQO1 (Novus #NB200-209, 1:1000).

### Human Tumor Specimens

FFPE oropharyngeal squamous cell carcinoma specimens were obtained and used in accordance with the Washington University in St Louis Institutional Review Board (IRB-201102323). Collection and use of FFPE oral cancer specimens was approved by the Stanford Institutional Review Board (IRB-10564). The human studies reported in this manuscript were carried out in accordance with the declaration of Helsinki. Genotyping of the oral cancer specimens was carried out by next generation sequencing and Sanger sequencing as reported in Guan *et al* (14).

### Sample Preparation for MS Analyses

Frozen cell pellets were lysed in an aqueous solution of 100 μl of 8 M urea, 75 mM NaCl, 50 mM Tris (pH 8.0), and 1 mM EDTA with addition of phosphatase and protease inhibitor cocktails (Halt, catalog no. 78429; 78420). Lysates were incubated on ice for 30 min with vortexing once every 5 min. Following high speed clearance protein was quantified by BCA (Thermo Fisher Scientific, catalog no. 23225). Samples were normalized to equal mass of protein and volume, and proteins were sequentially reduced in aqueous 5 mM DTT at 37 °C for 1 h and then alkylated in aqueous 50 mM 2-chloroacetamide at room temperature in the dark for 20 min. Samples were then diluted to 3 M urea with aqueous 50 mM Tris–HCl (pH 8.0) to prepare for digestion at 30 °C for 2 to 4 h with 20 mAU of lysyl endopeptidase (Wako Chemicals, 12902541) per 1 mg of protein. After further dilution to <2 M urea, trypsin (Promega, PR-V5113) was added at a 1:49 (wt/wt) enzyme-to-substrate ratio for overnight digestion at 37 °C. Following digestion, samples were brought to 1% formic acid (FA) by volume, high speed cleared at 21,000*g* at room temperature, and then desalted using 50-mg tC18 SepPak cartridges (Waters Technologies, WAT054960). Clean peptide was frozen, dried by vacuum centrifugation, and then reconstituted in a mass spectrometry–compatible loading buffer. Final peptide quantitation was determined *via* BCA but using a peptide digest standard (Thermo Fisher Scientific, catalog no. 23295).

Proteins were extracted from FFPE tissues and then digested as follows using a protocol based on Coscia *et al*. and Kohale *et al*. (42, 43). Surgical resections of HPV-subtyped oropharyngeal squamous cell carcinomas were obtained as 50-micron FFPE curls. Specimens were deparaffinized in 1.7 ml Eppendorf tubes with two sequential washes in 500 μl Xylenes at 56 °C and then rehydrated by washes in once each of 100, 95, 80, and 50% (v/v) ethanol in water. A lysis buffer of 2,2,2-trifluoroethanol and aqueous 300 mM Tris–HCL (pH 8.0) at 50% (v/v) was added to each sample. Curls were then ground ∼30 s each with a micro-pestle, snap frozen in liquid nitrogen, and then heated to 95 °C for 30 min. Subsequent probe sonication with a Model 120 Sonic Dismembrator (Thermo Fisher Scientific) at 70% amplitude in 10 cycles of 2 s on and 8 s off was followed by a second heating step at 95 °C for 90 min with occasional vortexing. Samples were then high speed cleared at 21,000*g* at room temperature, reduced, and alkylated as with the frozen cell pellets. Prior to digestion, each aliquot was concentrated by vacuum centrifugation to ∼50 μl and then brought to 500 μl with a 5% (v/v) 2,2,2-trifluoroethanol aqueous digestion buffer. Next, sequential lysyl endopeptidase and tryptic digestions were carried out as with the cell pellets assuming a 500 μg protein yield per sample. Samples were desalted by SDB-RPS spin columns (Affinisep, Spin-RPS-M.T1.96). Equilibration was with 200 μl acetonitrile (ACN) followed by 200 μl 0.5% FA in water. Samples were loaded in 1% TFA and then washed with 200 μl 0.2% FA in water and then by aqueous 200 μl 40% ACN in 0.5% FA. Peptides were eluted with a solution of 5% ammonium hydroxide, 15% water, and 80% ACN.

### Liquid Chromatography

Tryptic peptides were separated by reverse phase nano-HPLC using an Ultimate 3000 RSLCnano System (Thermo Fisher Scientific) coupled to a 25 cm × 75 um i.d. EASY-Spray HPLC column (Thermo Fisher Scientific) packed with 2 um C18 particles and heated to 40 °C. For peptide separation and elution, solvent A was 0.1% FA in water and solvent B was 0.1% FA in ACN. Samples were loaded by a user defined program for a 1 μl full-loop injection. For the cell line IS-PRM injections, the gradient was 2%B at 5 min, 4.3%B at 5.3 min, 8.0%B at 10.25 min, 10%B at 20 min, 16.5%B at 41 min, 19.2%B at 45.5 min, 22%B at 50.75 min, 54.4%B at 28 min, and 76.0%B at 56 min. Data-dependent acquisition (DDA) experiments were carried over a 118 min gradient: 2%B at 5 min, 19%B at 112 min, 38%B at 121 min, and 76%B at 123 min. For the FFPE IS-PRM injections, the gradient was 4%B at 1 min, 15.4%B at 31 min, 24.5%B at 46 min, and 98%B at 48 min. Each method included a wash step with three ramps between 2% and 98% solvent B followed by 24 min of re-equilibration at 2%B and 300 nl/min flow. During the gradients, the flow was 250 nl/min. For the cell line and FFPE tissue digests, 1 μg and 1.5 μg of endogenous peptide, respectively, were injected per run.

### Development and Analytical Validation of Targeted MS Assays

The targeted proteomic assays reported in this manuscript are Tier 2 level, which refers to analyses that use isotope-labeled internal standards for each analyte with the purpose of measuring relative protein abundances for nonclinical uses as described in Carr *et al*. (44).

#### Selection and Storage of SIL Internal Standard Peptides

For the HNSCC peptide library, SIL peptides were obtained in crude purity from Vivitide. These were reconstituted, combined to a nominal abundance of 300 nM/μl per peptide, aliquoted, and dried by vacuum centrifugation for storage. For each selected protein (see Results), three or more peptide representatives were chosen from the ProteomeTools database (45). When possible, we selected peptides between 7 and 16 amino acids in length and in order of decreasing priority, those not containing methionine, cysteine, or known phosphosites in the PhosphoSitePlus database (46). Under those constraints, we selected the most proteotypic peptides based on identifications in the ProteomicsDB database (47). The initial library included 288 peptides. Synthetic peptides missing the expected peak in their MALDI spectra or failing detection by at least five transitions in LC-MS/MS survey analyses were excluded from subsequent analyses, which left 236 peptides remaining. The supplementary material includes a catalog of these peptides. The Kinome SIL peptide library included 705 high-purity unmodified SIL peptides that were detectable in survey analyses.

#### MS Characterization of SIL Peptides

All MS data used in this manuscript were generated using an Orbitrap Eclipse Tribrid mass spectrometer (Thermo Fisher Scientific). Survey runs to characterize SIL peptides were carried out in a directed DDA mode with the injection of 150 fmol/peptide nominal abundance on-column as with all subsequent IS-PRM runs. Resolution is stated at 200 m/z. MS1 scan parameters were as follows: scan range, 300 to 1500 m/z; automatic gain control (AGC), 1.2e6; maximum injection time (maxIT), 50 ms; and orbitrap resolution, 120K. Up to 70 precursors from the inclusion list were subjected to MS2 scans in each cycle with a mass tolerance of 10 ppm. The quadrupole isolation width was set to 1 Th and higher energy collisional dissociation (HCD) fragmentation to an normalized collision energy of 35%. Orbitrap MS2 scans employed a scan range of 150 to 1700 m/z, AGC target at 5e5, maxIT of 10 ms, and an orbitrap resolution at 7.5k. In all subsequent methods, identification and quantitation of each SIL peptide was based on the six most abundant transitions in the survey analysis but excluding precursor, y_1_, y_2_, and b_1_ ions.

#### SureQuant Algorithm

SureQuant (Thermo Fisher Scientific) uses SIL internal standard peptides to direct acquisition of MS2 scans targeting the unlabeled, endogenous counterparts (41). In each cycle, the SureQuant algorithm looks for peaks in a high-resolution MS1 scan that match one or more precursors in the SIL library to within a given mass-to-charge tolerance. Upon detection of a SIL precursor exceeding an intensity threshold, SureQuant requests a “watch” MS2 scan targeting that precursor. If in the watch scan five or more transitions, selected from the survey runs, match their expected m/z to within specification, then SureQuant requests a “quant” MS2 scan targeting the endogenous precursor. The MS1 precursor mass tolerance was set to ± 5 ppm, and the MS2 transition tolerance set to ± 20 ppm. In downstream analyses, SIL peptide peak areas were calculated from their watch scans and endogenous peak areas from their quant scans. A retention time window for each precursor was set to ± 4 min of the survey run retention time. The maximum cycle-time was restricted to 3 s.

#### OIS-PRM Algorithm

We used the Thermo Scientific Tribrid instrument application programming interface (IAPI) to implement an OIS-PRM algorithm that differs from SureQuant in the following ways.

(1) OIS-PRM prohibits future quant scans for a precursor if after a minimum number of quant scans, one of the following three conditions holds.
  (i) The summed intensity of selected fragment ions for an SIL precursor is recorded for each scan. That intensity may fall below a user-defined percentage of the maximum observed for a given precursor during the current injection.
  (ii) The time since the first quant scan for a precursor may exceed an expected peak width threshold.
  (iii) A watch scan can fail to identify an SIL precursor with at least a minimum number of transitions. If this happens, there are two outcomes. First, if at least a minimum number of quant scans have been recorded for the precursor, then that precursor is excluded from future quant scans. Otherwise, the algorithm resets the running count of quant scans and future quant scans are not excluded.
(2) OIS-PRM enforces scan-order within each cycle by requesting scans at two points during each cycle in the following way. First, after the return of the MS1 scan, the IAPI requests any watch scans determined from the MS1 scan. Second, after completion of all outstanding watch scan requests, the IAPI tests whether each watch scan was successful. Success denotes a watch scan where at least a minimum number of the expected transitions are present. Then, the IAPI queues a watch and quant scan pair for each successful watch scan. Following a successful watch scan for a given precursor, the next MS1 scan may not trigger a watch scan for that precursor. Within a batch of requests, scans are always submitted in the following order of decreasing priority: first quant MS2 for a given precursor, quant MS2, MS1, watch MS2 triggered by a prior watch MS2, and watch MS2 triggered by an MS1. In each cycle, an MS1 is followed by all watch MS2 scans, which are followed by all quant MS2 scans. As a corollary of this scheme, there is a zero-cycle delay between the first MS1 detection of an SIL peptide and the first watch and quant scans for that precursor. supplemental Fig. S4 presents a schematic of the IS-PRM algorithm. While the instrument is waiting for scan requests from the IAPI, it repeatedly records default “no-op” scans (see “OIS-PRM Analyses”).

We ran OIS-PRM with the following parameters: minimum quant scans per peak of 10, expected peak width of 50 s, peak closeout intensity threshold at 15% of apex intensity, minimum number of transitions at 4, transition tolerance at ± 20 ppm, precursor tolerance at ± 5 ppm. The IAPI did not enforce an MS1 intensity threshold and instead required only detection of the two most abundant isotopes to trigger a watch scan. Default method scans take the lowest priority and only occur when the instrument is waiting for scan requests from the IAPI. The default method consisted of repeating linear ion trap MS1 scans. The OIS-PRM method is further detailed in supplemental Fig. S5.

#### IS-PRM Data Acquisition Parameters

For all OIS-PRM and SureQuant runs, Orbitrap MS1 scans used an AGC target of 1.2e6, resolution of 120k, and a maximum injection time of 50 ms. Fragmentation was carried out *via* HCD with a normalized collision energy of 30% and a precursor isolation width of 1 Th. All MS2 watch scans were carried out with an AGC target of 5e5, maxIT of 11 ms, and orbitrap resolution of 7.5k. Likewise, for the OIS-PRM experiment using kinome peptides, we specified an AGC target of 5e5, a maxIT of 160 ms, and an orbitrap resolution at 60k for all MS2 quant scans. For NRF2 panel experiments, these settings were 5e5, 246 ms, and 120K, respectively. For FFPE-derived samples, custom maxITs as high as 738 ms were specified for a handful of low abundance endogenous precursors as specified in the supplemental data. Linear ion trap MS1 no-op scans from the OIS-PRM method had an AGC target set to standard and scan speed set to turbo.

#### Peak Area Ratio Estimation

For both the standard SureQuant and OIS-PRM methods, text files were generated from an active IAPI instance recording all centroided MS2 scans with retention time and precursor m/z annotations. Custom python scripts were used to estimate peak area ratios (PARs) from these data as follows. Transition intensities were extracted with a width of 80 ppm for watch scan MS2s and 20 ppm for quant scan MS2s. If multiple peaks fell within a single extraction window, the one nearest the center of the window was used. Spectral contrast angles were used to identify and exclude noisy or interfered transitions in two ways. First, for each transition, two vectors of intensities from the light and heavy peptides were compiled over the integration bounds, and if the angle between those vectors exceeded $\frac{\pi }{8}$ radians, that transition was excluded from further analysis. Second, the three transitions with the highest summed peak area such that the spectral contrast angle between the SIL and endogenous peak areas did not exceed $\frac{\pi }{16}$ radians were used for quantification. This is illustrated in supplemental Fig. S7, and Gallien *et al*. have previously reported use of spectral contrast angles to detect interfered transitions for IS-PRM applications (37). The integration boundary was taken to be the longest streak of consecutive cycles that each contained both a watch and quant scan for the precursor. The longest streak could be broken by at most three consecutive cycles missing either or both of the watch and quant scans. The supplemental data report all chromatograms for each precursor and the subsets of transitions used for quantitation.

#### Normalization

We performed global extraction from PRM as proposed by Chambers *et al*. to normalize observed PARs based on the intensities of commonly identified peptides that are co-isolated with the targeted peptides (48). Raw files were converted to an open format (.mzML) using the ProteoWizard MSConvert tool (49). A python script removed linear ion trap no-op scans from the IAPI method.mzML files, and the trimmed files were then searched using the MetaMorpheus software (https://github.com/smith-chem-wisc/MetaMorpheus) with match between runs (50). “Deconvolute precursors” was enabled for the identification of co-isolated precursors. Background peptides commonly identified between all runs were then used to calculate the median normalization factors as described by Chambers et al. (48). Peptides corresponding to targeted proteins were excluded from the analysis before normalization.

#### Peptide Imputation and Summarization to Protein

PARs for each peptide corresponding to a given protein were summarized to protein-level abundances by taking their geometric mean. We assumed that peptides derived from the same protein were correlated in abundance. Accordingly, we used a k-nearest neighbors imputer as implemented scikit-learn to impute missing peptide values given the nonmissing values for peptides corresponding to the same protein (51). For a given experiment, a peptide was excluded from analysis if it was missing from greater than one-half of samples from which at least one other peptide from the same protein was quantified. When all peptides were missing for a given protein and injection, the protein was considered missing.

### Label-Free MS Analyses

For DDA experiments for the A549 cell line and HPV positive tumors, MS1 scans were carried out with a resolution of 120k, an AGC target of 1.2e6, and scan range of 375 to 1500 m/z. Dynamic exclusion was for 60 s. Fragmentation was performed for charge states between 2 and 7 inclusive. The HCD-normalized collision energy was set to 32%. For MS2 scans in the linear ion trap, the quadrupole isolation window was set to 0.7 Th, the maximum IT to 50 ms, and the AGC to standard. The cycle time was set to 3 s.

### Database Searching and Peptide Identification

For label-free analysis of the A549 cell line and HPV-positive tumors, the.raw files were searched using MaxQuant version 2.0.3.0 against the UniProt human proteome (Swiss-Prot + Trembl) in addition to the default MaxQuant contaminants with an false discovery rate of 1% (52). The precursor search tolerance was 4.5 ppm and the fragment search tolerance was 0.5 Da. For the cell lines and FFPE tissues respectively, these were downloaded on September 16, 2021 and February 18, 2023 and contained 78,120 and 79,038 sequences. Methionine oxidation and N-terminal acetylation were searched as variable modifications. Cysteine carbamidomethylation was fixed. Lysine methylation and both N-terminal and lysine formylation have been reported as formalin fixation artifacts and were included as variable modifications for the FFPE-derived samples (53, 54). Quantification was by MaxLFQ and without match between runs (55).

### Experimental Design and Statistical Rationale

Throughout, correlations were assessed by Spearman’s rank correlation, and Mann-Whitney U tests were used to compare continuous variables sampled from two populations. We applied hierarchical Bayesian analyses to model the expression of NRF2 target proteins for the 21 cell lines and then for the NRF2-genotyped oral cavity tumors as described in the supplemental methods.

Oral squamous cell carcinoma–derived cell lines, SCC90, SCC152, and SCC154, stably expressing NRF2 E79Q and their parental cell lines were each cultured in separate 10 cm plates and harvested on the same day. This was done twice, once for Western blot and a second time for IS-PRM. Cell lines were cultured each in biological duplicate and the mean value for each protein across both replicates used in the final analyses. The NRF2 status of the cell lines was determined by hierarchical clustering using Euclidian distance and with the Ward method.

Finally, NRF2 scores represent the position of each tumor along the first principal component of the data. For the Clinical Proteomic Tumor Analysis Consortium (CPTAC) data, the principal component analysis (PCA) was performed on the 13 NRF2 target proteins from the pilot SIL peptide array and shown. For the analysis of oropharyngeal and oral squamous cell carcinomas, the PCA analyses were performed on the NRF2 target proteins that were differently expressed between the NRF2 active and inactive cell lines as shown.

## Results

### Benchmarking an Optimized IS-PRM Method

**Fig. 1:**
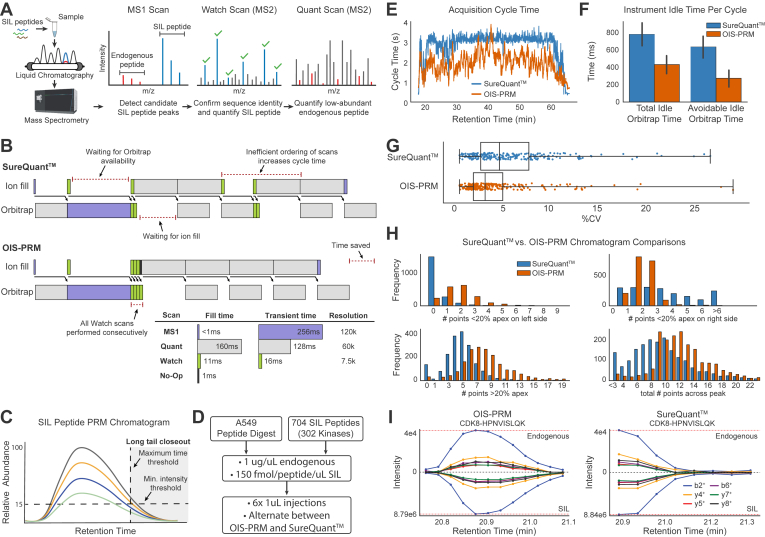
**OIS-PRM enabled efficient targeted proteomic data acquisition.***A*, schematic of the SureQuant (Thermo Fisher Scientific) IS-PRM acquisition algorithm. Stable isotope-labeled (SIL) peptides corresponding to each endogenous targeted peptide are spiked into each sample. The heavy SIL peptides are highly abundant, direct efficient data acquisition of endogenous light peptides, and serve as internal standards for quantification. MS1 detection of an SIL peptide triggers an MS2 “Watch” scan for the SIL peptide. When a “Watch” scan confirms an SIL peptide by the presence of predetermined fragment ions, SureQuant triggers a “Quant” MS2 scan targeting the endogenous counterpart. Signal intensity from each endogenous peptide is normalized to its corresponding SIL internal standard peptide. *B*, example of instrument utilization on a Tribrid mass spectrometer using SureQuant or our optimized internal standard PRM (OIS-PRM) method. OIS-PRM contiguously performs all “Watch” and “Quant” scans. *C*, illustration of thresholds used to stop data acquisition of long elution tails. “Watch” and “Quant” scans are not acquired on a target after the summed fragment ion intensity for the SIL peptide falls below a proportion of the maximum observed for that SIL peptide or after a maximum time since its initial detection. *D*, experimental design for the comparison of SureQuant and OIS-PRM methods in the subsequent panels. *E*, cycle time plot for representative injections. SureQuant cycle times were capped at a maximum of 3 s. *F*, comparison of Orbitrap idle time. Avoidable idle time subtracts the transient of the MS1 scan from the total. *G*, comparison of coefficients of variation (CV) for endogenous peptides over three replicate injections. *H*, number of peptides whose quantified chromatograms satisfied the indicated constraints for all three replicate injections. *I*, chromatograms for the same peptide in back-to-back injections using OIS-PRM and SureQuant. IS-PRM, internal standard–triggered parallel reaction monitoring.

Two targeted proteomics methods, IS-PRM and its commercial implementation called SureQuant, achieve sensitive and reproducible quantification of peptides by monitoring spiked-in SIL peptides to direct efficient data acquisition. These internal standard peptides co-elute with their endogenous counterparts so that IS-PRM uses the each SIL peptide to trigger time-intensive and quantitative “quant” scans targeting the corresponding endogenous peptide. Specifically, quant scans of the endogenous peptide are triggered by fast “watch” scans which detect and identify the highly abundant SIL peptide. Because of this efficient use of instrument time, IS-PRM enables quantification of more proteins in a single analysis than a standard PRM method (Fig. 1*A*) (37). However, through our research with SureQuant, we observed that inefficient scan scheduling on Tribrid mass spectrometers resulted in excess Orbitrap idle time (Fig. 1*B*). Using the Thermo Scientific Tribrid IAPI, we implemented an OIS-PRM method that postpones quant scans until the completion of all SIL-detection watch scans (Fig. 1*B*). Additionally, we hypothesized that avoiding quant scans during the long tail of a peptide elution profile would free up instrument time without compromising quantitative accuracy; therefore, we added thresholds for minimum relative intensity and maximum elution time (Fig. 1*C*). Last, we reordered quant scans to prioritize newly detected peptides and capture the start of their elution profiles.

To evaluate OIS-PRM performance, we ran six injections of an A549 lung cancer cell line digest and alternated between SureQuant and OIS-PRM (Fig. 1*D*). The SIL library included 704 kinase-associated peptides representing 302 kinases. These were analyzed over a 50 min liquid chromatography gradient. OIS-PRM reduced the median cycle time from 3.1s to 1.8s (Fig. 1*E*); efficient scan ordering contributed 360 ms per cycle to this difference (Fig. 1*F*). With maximum time and intensity thresholds, the number of SIL peptides with at least seven points across the peak improved from 173 to 252 and median CV from 4.6 to 3.2 percent for OIS-PRM compared to SureQuant (Fig. 1*G*). Overall, OIS-PRM and SureQuant quantified 264 and 259 peptides, respectively, with CVs less than 20%. OIS-PRM sampled more points per peak, missed fewer peak fronts, and oversampled from peak tails less often (supplemental Fig. S1, *H* and *I*). Finally, we compared OIS-PRM to a standard DDA method, frequently used in our laboratory, which quantifies peptides based on the intensity of MS1 peaks. Over three replicate injections, the OIS-PRM method quantified 172 out of 302 kinases with a CV less than 20% (supplemental Fig. S1). Although DDA identified 4680 protein groups on average, only 47 kinases were quantified with a CV under 20%. Therefore, the DDA method could not quantify even half as many kinases as OIS-PRM despite that the untargeted method used a gradient length of 118 min, nearly double that of the OIS-PRM method.

### Development of an Internal Standard Peptide Array for HNSCC

**Fig. 2:**
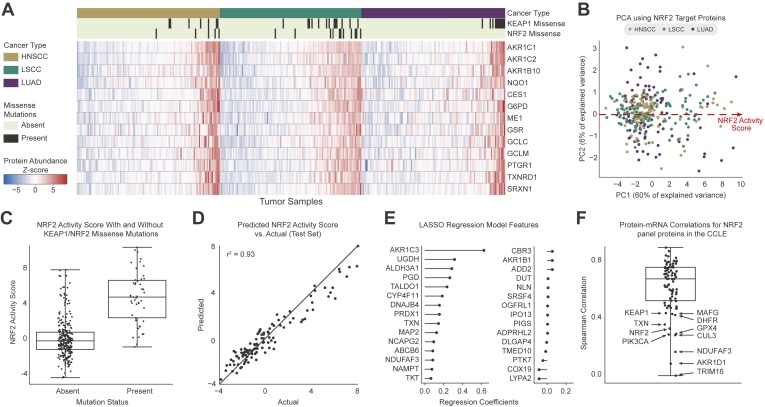
**Combined analysis of three Clinical Proteomics Tumor Analysis Consortium cohorts showed that a strongly correlated subset of proteins could predict NRF2/KEAP1 genotype.***A*, protein expression of thirteen NRF2 targets in CPTAC cohorts for HNSCC, LSCC, and LUAD. NRF2 mutations distant from the ‘DLG’ or ‘ETGE’ motifs were excluded from the count. *B*, PCA plot of the first two principal components for the CPTAC data by the thirteen NRF2 target peptides. The NRF2 activity score refers to the position along the first principal component of the data. *C*, distribution of NRF2 scores for the combined CPTAC cohorts split based on the presence or absence of missense mutations in KEAP1 or NRF2. *D*, a LASSO regression model trained to predict the NRF2 scores for tumors given the 6133 proteins not included in the original set of thirteen NRF2 targets. The model was trained by 10-fold cross validation on a training set of 2/3 of the data (218 tumors). Predicted NRF2 scores are plotted against the true NRF2 pathway scores for an independent test set containing one-third of the data (109 tumors). *E*, feature weights for the regression model in (*C*). *F*, protein to mRNA correlations for the NRF2 IS-PRM panel proteins across the cancer cell line encyclopedia. CPTAC, Clinical Proteomic Tumor Analysis Consortium; HNSCC, head and neck squamous cell carcinoma; IS-PRM, internal standard–triggered parallel reaction monitoring; PCA, principal component analysis.

Aberrant NRF2 activity prognosticates resistance to radiation and chemotherapy in cancers of the lung and upper airway. Therefore, to empower the clinical potential of OIS-PRM, we developed an NRF2 and HNSCC-specific SIL peptide catalog. This resource includes 227 peptides that represent 90 proteins: 68 NRF2-interacting proteins or transcriptional targets; 10 immuno-oncology markers that include immune checkpoint proteins, cytokines, T-cell surface markers, and immuno-oncology markers; 8 known SCC tumor suppressors and oncogenes; HPV E6 and E7, GAPDH, and DHFR. To develop the NRF2-activity SIL panel, we began with 23 well-established NRF2 targets and NRF2-interacting proteins (supplemental Table S1). For expansion, we proteogenomically analyzed the CPTAC LUAD, LUSC, and HPV(−) HNSCC cohorts, which frequently contain NRF2 pathway–activating mutations (supplemental Fig. S2) (56, 57, 58). We sorted the tumors by the abundance of the 13 NRF2 target proteins that were expressed in all of the tumors (Fig. 2*A*). Principal component analysis compressed these data into a single “NRF2 activity score” that captured 60% of the data variance (Fig. 2*B*). Overall, NRF2/KEAP1-mutated tumors reported higher NRF2 activity scores than tumors lacking mutations (Fig. 2*C* and supplemental Fig. S3). However, many tumors with mutations showed low expression of NRF2 target genes and conversely, many KEAP1 and NRF2 WT tumors overexpressed NRF2 targets. As such, assays that classify NRF2 activity in tumors based on genotype alone will suffer from high false positive and false negative rates (Fig. 2*C*).

We therefore used the CPTAC data to identify additional proteins useful for monitoring the NRF2 pathway. A LASSO regression was trained on >6000 proteins not among the initial 13 NRF2 targets to predict the NRF2 activity scores across the CPTAC cohorts (Fig. 2*D*). We found that a mere 30 proteins with non-zero coefficients in the model could accurately predict the NRF2 scores (Fig. 2*E*). Literature evidence for most proteins with non-negligible coefficients supported their status as NRF2 targets. We included 17 of these in the final SIL peptide array as described in the supplemental methods and tables (supplemental Tables S1 and S2). Finally, we inspected the mRNA to protein correlations within our NRF2 panel using the Cancer Cell Line Encyclopedia (31). While most NRF2 transcriptional targets encode proteins that correlated well with their respective mRNA, key regulatory proteins NRF2, KEAP1, MAFG, CUL3, and TRIM16 correlated poorly (Fig. 2*F*). Similarly, many immune checkpoint proteins correlated poorly with their mRNA abundances, which agree with recent reports (29, 30, 31, 59).

### Validation of the NRF2 Pathway in Cell Lines

**Fig. 3:**
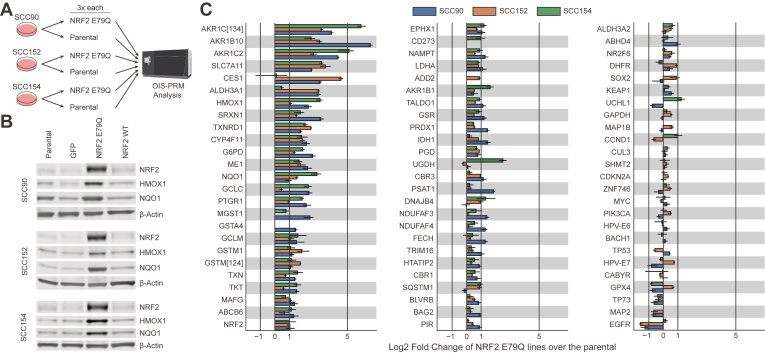
**Oral squamous cell carcinoma cell lines that stably overexpressed a cancer-derived NRF2 variant likewise overexpressed NRF2 targets at the protein level.***A*, parental oral squamous cell carcinoma cell lines stably expressing WT NRF2 and derived cell lines stably expressing NRF2 E79Q were grown in three replicates each, harvested on the same day, and analyzed by OIS-PRM. *B*, protein expression measured by Western blot from whole-cell lysates of cell lines derived from human oral squamous cell carcinomas. Blots include parental cell lines and those stably expressing GFP, NRF2 E79Q, and NRF2 WT. *C*, parental and NRF2 E79Q cell lines as analyzed by OIS-PRM. Error bars represent the minimum and maximum log_2_ fold change between pairs of parental and NRF2 E79Q replicates.

To assess whether our IS-PRM assay could quantify NRF2 pathway activation, we engineered HPV(+) HNSCC cell lines SCC90, SCC152, and SCC154 to stably express NRF2^E79Q^, a common cancer-associated activating NRF2 mutation (Fig. 3*A*) (60). Stable expression of NRF2^E79Q^ protein in each cell line induced the expression of NRF2 and two of its canonical targets, NQO1 and HMOX1 (Fig. 3*B*). OIS-PRM analyses of the six cell lines revealed that NRF2 activation induced protein expression of NRF2 target genes over parental controls (Fig. 3*C*).

**Fig. 4:**
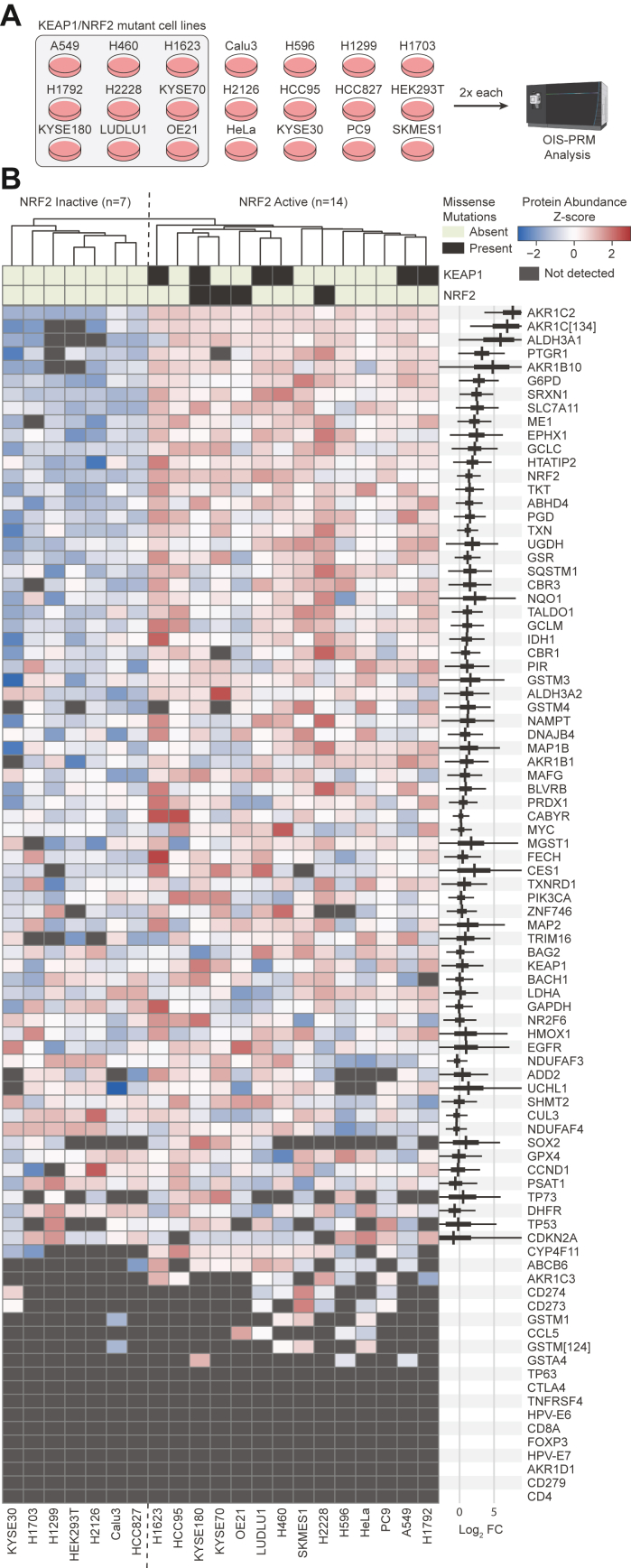
**OIS-PRM analysis of 21 cancer cell lines distinguished between NRF2 active and inactive cell lines.***A*, twenty-one cancer cell lines were cultured in biological duplicate, harvested, and subject to OIS-PRM using an HNSCC-specific SIL peptide array. *B*, results of the experiment described in (*A*). Protein abundances were averaged for each replicate and then data were row-normalized by Z-scores and hierarchically clustered. On the *right-hand-side*, the *thick black bands* contain 95% of the posterior density of the mean logarithmic fold change between the active and inactive clusters. The narrow *gray* bands contain 95% of the posterior predictive density for the logarithmic fold change in expression between an NRF2 active over an NRF2 inactive cell line. HNSCC, head and neck squamous cell carcinoma; SIL, stable isotope labeled.

For additional testing, we applied OIS-PRM method to a collection of 21 cell lines with known *NRF2/KEAP1* genotype and activity status (Fig. 4*A*). Hierarchical clustering placed all cell lines with *NRF2* or *KEAP1* mutations in the same cluster (Fig. 4*B*). In agreement with the CPTAC cohorts (Fig. 2*A*), several cell lines lacking a causative mutation overexpressed NRF2 targets. We modeled expression of NRF2 target proteins using a hierarchical Bayesian analysis and found that the cluster of cell lines containing all NRF2/KEAP1 mutations overexpressed well-validated NRF2 targets, such as NQO1, GCLC, and SLC7A11(XCT), compared to the NRF2 inactive cluster. The abundance of NRF2 itself, however, did not perfectly discriminate between the active and inactive cell lines. The posterior density of the logarithmic fold change parameter for GAPDH concentrated around zero and suggested good data normalization.

### OIS-PRM Analysis of HNSCCs

**Fig. 5:**
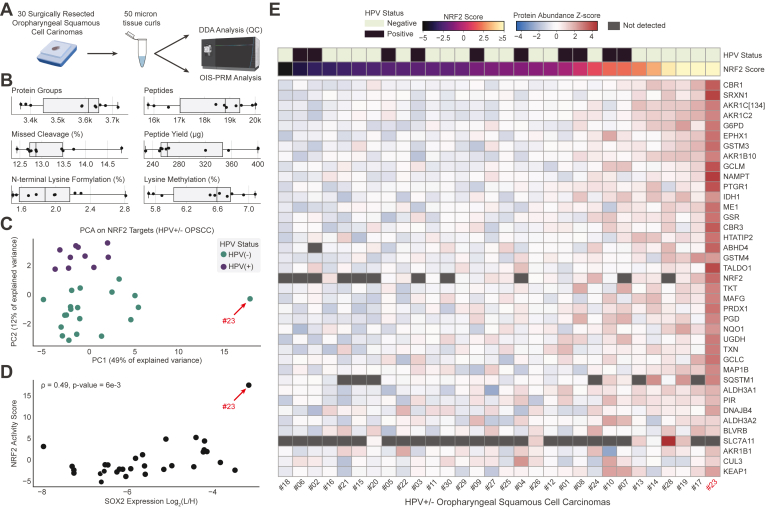
**OIS-PRM analysis of FFPE oropharyngeal squamous cell carcinomas revealed a large dynamic range of NRF2 target expression.***A*, schema describing the collection and analysis of 20 HPV(−) and 10 HPV(+) oropharynx tumors. Protein was extracted from 50-micron curls cut from FFPE tumor blocks and subject to OIS-PRM. *B*, summary statistics for data-dependent acquisition proteomics on the 10 HPV(+) tumors lysine methylation and N-terminal lysine formylation are common artifacts of formalin fixation. *C*, PCA plot from protein abundances of differently expressed NRF2 target proteins as measured by OIS-PRM. *D*, scatterplot of NRF2 activity scores and SOX2 protein abundance for each tumor. SOX2 abundance is reported as the Log2-transformed geometric mean of the peak area ratios for the two representative peptides: APCQAGDLR and LLSETEK. The NRF2 score refers to the position along the first principal component from (*C*). *E*, heatmap of OIS-PRM data with row-normalized Z-scores. HPV, human papilloma virus; PCA, principal component analysis.

**Fig. 6:**
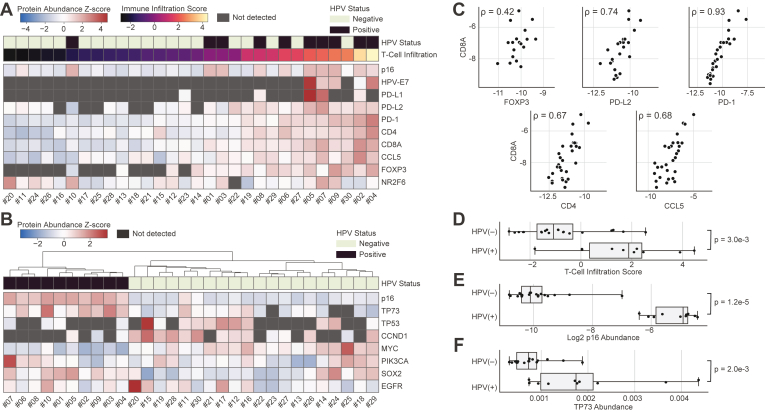
**OIS-PRM analysis of FFPE oropharyngeal squamous cell carcinomas reveals HPV-specific differences in the expression of T-cell markers and oncogenes.***A* and *B*, expression of immuno-oncology markers and cancer drivers by OIS-PRM for the same oropharynx tumors as in Figure 5. *C*, spearman correlations between the immuno-oncology markers shown in *A*. *D*–*F*, immune infiltration score, p16 abundance, and TP73 abundance split by HPV status. A PCA analysis of the tumors in (*A*) using PD-1, CD4, CD8α, CCL5, and FOXP3 expression as features was used to calculate the T-cell infiltration scores as the position of each tumor along the first principal component. *p*-values were calculated by a two-sided Mann-Whitney U-test. HPV, human papilloma virus; PCA, principal component analysis.

After establishing OIS-PRM in cultured cell models, we next tested it across two sets of archived FFPE HNSCC tumors: (1) 10 HPV(+) and 20 HPV(−) oropharyngeal squamous cell carcinomas collected as 50 μm curls (Fig. 5) and (2) punch biopsies from 19 HPV(−) oral squamous cell carcinomas including 11 NRF2 WT tumors and 8 tumors with NRF2^E79Q^ or NRF2^E79K^ activating mutations (Fig. 6). After testing and optimizing a protocol for protein extraction from FFPE, we evaluated protein quality by DDA-MS on 10 HPV(+) oropharyngeal squamous cell carcinomas FFPE curls (Fig. 5, *A* and *B*). On average, each 50 μm curl yielded 300 μg of protein and 18,200 peptides mapping to 3600 protein groups (Fig. 5*B*). These yields and overall peptide characteristics were similar to those of prior FFPE proteomic studies (42, 43, 61, 62).

OIS-PRM analysis of the 30 FFPE curls from HPV(+) and HPV(−) oropharyngeal squamous cell carcinomas revealed expected and novel protein correlations. As with the CPTAC cohort, the first principal component served as an NRF2 score, and it explained fifty percent of the variance (Fig. 5*C*). This NRF2 score positively correlated with SOX2 protein abundance (Spearman r = 0.49, *p*-value = 0.006, Fig. 5*D*). Unexpectedly, we also observed that the second principal component NRF2 score perfectly separated the HPV(+) from the HPV(−) tumors. One of the thirty tumors substantially overexpressed NRF2 targets relative to the others. Indeed, this tumor expressed six of the NRF2 targets (CES1, CYP4F1, GSTM3, ARK1C1/3/4, AKR1C2, SRXN1, and PTGR1) more than 16-fold above their respective mean expressions for the entire cohort and 24 proteins more than 4-fold above their means (Fig. 5, *C* and *E*).

In the same OIS-PRM experiment, we quantified immuno-oncology biomarkers and cancer drivers (Fig. 6, *A* and *B*). The abundances of T-cell–associated proteins, CD8α, FOXP3, PD-L2, PD-1, CD4, and CCL5, correlated with one another across the cohort (Fig. 6*C*). Notably, PD-1 had a near-perfect rank correlation with the cytotoxic T-cell maker, CD8α, and with the exception of FOXP3, these rank correlations were as strong or stronger than for the typical protein and its mRNA in the CCLE (31). Using a principal component analysis, we derived a T-cell infiltration score and found that HPV(+) tumors displayed significantly higher T-cell infiltration than HPV(−) tumors (Fig. 6*D*). PD-L1 and PD-L2 were detected in 16% and 80% of the tumors, respectively. Nearly all tumors expressed the transcriptional factor and immune checkpoint, NR2F6, at detectable levels (63, 64).

Protein expression of p16 is a commonly used surrogate for HPV infection; direct MS-based detection of endogenous HPV has not previously been established. OIS-PRM detected the E6 and E7 proteins in the HPV(+) oral squamous cell carcinoma cell lines, SCC90, SCC152, and SCC154 (Fig. 3*C*). Across the oropharyngeal squamous cell carcinomas tumor cohort, we detected E7 in five of the 10 HPV(+) tumors and in none of the HPV(−) tumors (Fig. 6*A*). As expected, p16 expression separated HPV(+) from HPV(−) tumors (Fig. 6*E*). HPV(+) tumors also significantly overexpressed TP73 compared to HPV(−) tumors, which agrees with a previous report (Fig. 6*F*) (65).

**Fig. 7:**
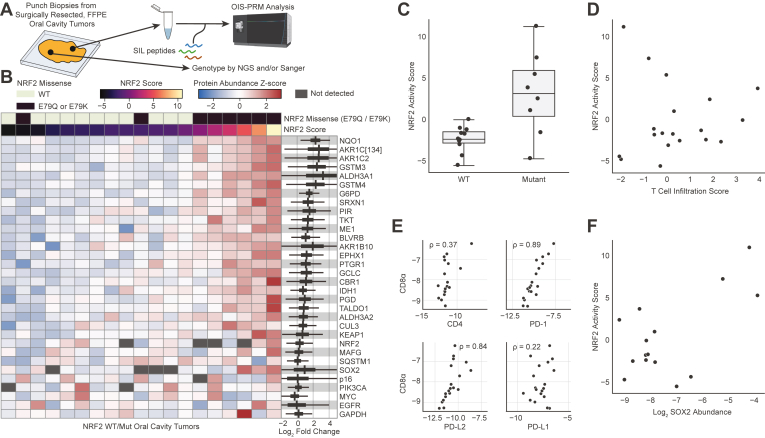
**OIS-PRM analysis of oral squamous cell carcinomas showed concordance between NRF2 genotype and target gene expression.***A*, schema describing the collection, genotyping, and OIS-PRM analysis of oral cavity tumors. Protein was extracted from punch biopsies of FFPE tumor blocks and subject to OIS-PRM. Tumors were either NRF2 WT, NRF2 E79Q, or NRF2 E79K. *B*, expression of NRF2 target proteins and others by OIS-PRM. On the *right-hand-side*, the *thick black bands* contain 95% of the posterior density of the mean logarithmic fold change between the active NRF2 Mut and inactive NRF2 WT tumors. The narrow *gray* bands contain 95% of the posterior predictive density for the logarithmic fold change in expression between an NRF2 active over an NRF2 inactive tumor. *C*, NRF2 activity scores for the NRF2 WT and mutant (E79Q or E79K) tumors. *D*, scatterplot of immune infiltration scores and NRF2 activity scores for each tumor. Scores were calculated by PCA as in Figures 6 and 5 respectively. *E*, spearman correlations between the immuno-oncology markers. *F*, scatterplot of NRF2 activity scores and SOX2 protein abundance for each tumor. PCA, principal component analysis.

In addition to the HPV(+) and HPV(−) oropharyngeal squamous cell carcinomas, we also tested OIS-PRM on FFPE tumor punches from a cohort of 19 HPV(−) oral squamous cell carcinomas that were genotyped for NRF2 (Fig. 7*A*). These included eight *NRF2*^*E79Q*^ or *NRF2*^*E79K*^ activating/mutant tumors and 11 NRF2 WT tumors. Of the eight NRF2 mutant tumors, six strongly expressed NRF2 target genes. Several NRF2-target proteins were expressed at greater than 4-fold in NRF2 mutant than WT tumors, including NQO1, AKR1C2, GSTM3, GSTM4, and ALDH3A1 (Fig. 7, *B* and *C*). We further examined T-cell markers and found that their expression did not correlate with NRF2 activity (Fig. 7*D*). Nevertheless, correlations between T-cell markers and immune checkpoint proteins were often strongly positive just as with the oropharyngeal cohort (Fig. 7*E*). Finally, similar to the HPV cohort of oropharyngeal squamous cell carcinomas, SOX2 abundance and NRF2 activity trended to a positive correlation (Fig. 7*F*). This correlation supports a prior report showing that NRF2 activation associates with SOX2 amplification in squamous cell carcinomas (33).

## Discussion

This work presents an optimized targeted proteomics method called OIS-PRM and an SIL peptide library that may be valuable for basic, pre-clinical, and clinical research. Within the clinical arena, biomarker assays are needed in HNSCC to predict patient response to RT. Radiation functions as the core of therapy in locally advanced HNSCC either as definitive treatment with chemotherapy or following surgery and despite significant improvements in radiotherapy, patients with advanced disease still face poor outcomes. We and others recently reported that NRF2-activating genotypes predict poor response to RT, as quantified by locoregional failure following RT-based therapy (13, 14). The resulting assertion, which remains to be clinically implemented, is that HPV(−) HNSCC that are NRF2-inactive should receive standard of care radiation. Conversely, patients with NRF2-active tumors should consider alternative modalities to RT when appropriate or more aggressive therapeutic regimens. In addition to the primary treatment setting, recurrent HPV(+) tumors frequently harbor mutant/active NRF2 alleles (66); therefore, patients with recurrent HPV(+) cancer should undergo screening for NRF2 signaling if RT is to be considered as part of their treatment when other appropriate treatment options exist. For basic and translational cancer research, OIS-PRM provides a powerful multiplexed protein quantitation assay that if implemented as a shared resource would be cost effective and empowering. For example, though the FDA has not approved NRF2 inhibitors, future clinical trials for any such drugs could use OIS-PRM as a mechanistic biomarker and to stratify patients for trial consideration.

In addition to the value of NRF2-centered biomarkers, OIS-PRM–enabled protein-level quantitation of T-cell infiltration, and immune checkpoint proteins may be a useful adjunct to predict patient response to anti-PD1 therapies. Current predictive biomarkers for anti-PD1 therapy response include antibody staining for PD-L1, tumor mutational burden, and an mRNA expression–based IFN-gamma signature (16, 18, 19). OIS-PRM with an optimized SIL peptide library may provide some ancillary benefit to these methods, but at present, the strong colinearity between most of the immuno-oncology markers studied in this work would limit the predictive potential of our assay. Future expansion of our SIL peptide catalog will include additional immune checkpoint proteins, cytokines, chemokines, and markers for subtypes of innate immune cells that suppress antitumor immunity.

Patients with HPV(+) oropharyngeal squamous cell carcinoma tend to have good oncologic outcomes overall and this has spurred an interest in de-intensifying adjuvant and definitive radiation dosing for these patients. To date, no standard of care de-intensification regimen has emerged, in part because many studies have shown a reduction in locoregional and distant control with de-intensified radiation (67). Part of the problem may be that aside from clinical and pathologic staging, imaging, and smoking history, exceedingly few trials utilize tumor biology to stratify those patients at increased risk of recurrence (68). One study reported significantly reduced expression of T-cell markers in HPV(+) tumors that would eventually recur, compared to those that did not recur (69). Our OIS-PRM data reveal a small fraction of HPV(+) tumors that have low T-cell infiltration, comparable to that of typical HPV(−) tumors. Therefore, a rapid assay capable of reporting an NRF2 signaling score as well as a T-cell infiltration score may be a meaningful biomarker to predict radiation resistance and better stratify patients for de-intensification of clinical trials.

However, our results and analysis of publicly available CPTAC proteomics data suggest that neither genotype nor the expression of any single protein can accurately predict NRF2 pathway activity. Genotype-phenotype annotations for cancer-derived mutations remain sparse—particularly for tumor suppressor genes—and thus mutation-based classifiers often suffer high false discovery rates. Accordingly, it is difficult to predict the functional effects of KEAP1 mutation on NRF2 transcriptional activity (15). We found that protein expression NRF2 targets efficiently separated NRF2-active from NRF2-inactive tumors (Fig. 2, *A*–*C*), but not all of these targets are equally diagnostic for NRF2 activity. We therefore modeled the expression of each NRF2 target in our SIL peptide library in both cell lines and oral cavity tumors to quantify the extent and consistency to which NRF2 signaling drove the expression of each target (Figs. 4*B* and 7*B*). Notably, HMOX1 ranked poorly among all NRF2 targets in the panel despite its widespread use as a favored NRF2-activity marker (70).

RNA biomarkers and protein biomarkers independently offer great value for personalized medicine. With the rapid technological and computational advancements in MS, protein-based assays are approaching the comprehensiveness of genomic assays. Many features of proteins make them superior to mRNA-based biomarker assays, not the least of which are the complicated mechanisms governing the abundance of mRNA to its protein product (59, 71). We found that for NRF2 target genes, the protein-to-mRNA correlations are moderate to strong, such that transcript abundances do well to distinguish between the NRF2 active and inactive cases (Fig. 2*F*) (31). However, for other proteins in our catalog such as NRF2, KEAP1, PD-L1, PD-L2, and various immune checkpoint proteins and cytokines, correlations between the mRNA and respective proteins are weak or lacking in validation (29, 31, 72).

Our OIS-PRM analysis of HNSCC tumor samples revealed varied NRF2 activity. From a small cohort of 30 HNSCC oropharynx tumors, we identified a single HPV(−) tumor with exceptionally high abundance of NRF2 target proteins (Fig. 5). In addition, several HPV(−) tumors demonstrated moderately elevated NRF2 scores, perhaps owing to nongenomic mechanisms of pathway activation such as competitive KEAP1 inhibition or NRF2 copy number amplifications (15). Whether this intermediate NRF2 activation impacts responsiveness to RT remains to be seen, but future analysis of appropriately sized training and validation cohorts could reveal a threshold of clinical relevance. Subsequent analysis of a separate cohort of NRF2-genotyped oral cavity tumors further confirmed that targeted proteomics can identify NRF2 active tumors and quantify immune checkpoint proteins and cancer drivers (Fig. 7). However, two of the eight tumors harboring NRF2 E79Q or E79K alleles did not overexpress NRF2 targets at the protein level. We hypothesize that spatial heterogeneity within each tumor between the genotyped punch and the independent punch taken for proteomics could explain this discrepancy.

Our data also present several unexpected observations pertaining to NRF2-driven immune-suppression, a correlation between the NRF2 and SOX2 oncogenes, and NRF2 activation in an HPV(+) background. First, given recent publications, we expected NRF2 activation to inversely correlate with T-cell infiltration (18, 33, 34). Our data do not support this hypothesis. However, the literature strongly shows that NRF2 activity correlates with resistance to anti-PD1 drugs, drives expression of PD-L1, and supports polarization of tumor-infiltrating leukocytes towards immunosuppressive functions (14, 18, 32, 36). Therefore, it is possible that NRF2 mediates immune suppression by modulating the infiltration and function of innate immune cells rather than the abundance of T-cells at the primary tumor site. Indeed, we recently observed in mice that NRF2 activation within allogenic-grafted HNSCC tumors polarized infiltrating monocytes from an M1 towards an M2 phenotype and correlated with increased abundance of myeloid-derived suppressor cells (14). Likewise, overexpression of an NRF2 target, GPX2, in a different mouse model of oral cancer results in M2 skewing and an increase in myeloid-derived suppressors but with a reduction in T-cell infiltration (14, 34). Notably, the sample size in our study is limited, thus weakening statistically meaningful observations with respect to T-cell infiltration. Secondly, Harkonen *et al*. recently observed positive correlation between SOX2 copy number and NRF2 transcriptional signature (33). We also observed co-expression between the SOX2 and NRF2 oncogenes and believe this association merits further investigation. Finally, we observed that the second principal component of the NRF2 proteins separated HPV(+) from HPV(−) tumors, suggesting that NRF2 differently activates its target genes in an HPV(+) compared to an HPV(−) background.

In addition, several discussion points on the development of OIS-PRM are warranted. OIS-PRM differs from SureQuant and current state-of-the art methods primarily in that it efficiently orders scans within each scan cycle and monitors peptide elution in real-time to avoid acquiring uninformative scans during long peak tails. Prior art recommends rapid data acquisition to ensure capture of 6 to 10 data points for each peptide analyte (41, 73). However, this heuristic rule might apply differently depending on whether quantification relies on raw peak areas or on ratios with internal standards. In theory, a single measurement should reflect the relative abundances of an SIL peptide and its endogenous counterpart, with additional scans minimizing the effects of noise and variability. TMT-labeling experiments operate on this principle and quantify peptides by the relative abundances of reporter ions in as few as one MSn scan. Accordingly, while OIS-PRM increased the number of peptides quantified with at least seven points and decreased median CVs, it failed to quantify more peptides with a CV of less than 20%. When using internal standards, however, dense chromatogram sampling enables alignment of SIL and endogenous chromatographic profiles. Poor correspondence of light and heavy counterparts reveals interfered or noisy transitions unsuitable for quantification, with the absence of aligned transitions serving as a pseudo limit of detection. Therefore, this work and others describe spectral contrast angle metrics to measure similarity between light and heavy peptides (37, 74). We propose that a 1-cycle delay between MS1 detection and the subsequent watch scan could explain why the SureQuant method frequently missed peak fronts (Fig. 1*I*). Because of these aforementioned advantages, OIS-PRM will enable the use of even larger SIL peptide arrays of up to 700 peptides and thereby empower proteomic interrogation of tumor biology and personalized medicine.

Finally, we opted to use a custom analysis pipeline to process PRM data in order to optimize data analysis specifically for triggered PRM experiments. Doing so had several advantages; for example, our reported pipeline used different analysis parameters for watch and quant scans as these scan types differ in their acquisition parameters. In addition, we calculated spectral contrast angles between the SIL and endogenous peptides within each injection rather than between the endogenous peptide and a library reference spectrum from a prior experiment. Lastly, access to the raw data allowed for fine control over data visualization. We generated chromatogram mirror plots for easy visual alignment between the SIL and endogenous chromatograms and then combined all chromatograms into a single portable document format file for each RAW file.

## Data Availability

The mass spectrometry data files supporting the findings of this study are available in the PRIDE database at ProteomeXchange under the project identifiers PXD041162, PXD041163, and PXD042949. The data analysis pipelines as Jupyter Notebooks and python scripts in addition to peptide chromatogram plots and quantitation tables are available on Figshare under the project name 'Targeted Proteomic Quantitation of NRF2 Signaling and Predictive Biomarkers in HNSCC': https://doi.org/10.6084/m9.figshare.22634482.v1. These peptide and protein level data are summarized in supplemental Tables S4 and S5 respectively. Skyline files for viewing the PRM data are publicly available on the Panorama Public website at: https://panoramaweb.org/arn2Sp.url

## Supplemental data

This article contains supplemental data (45, 46, 51, 75, 76, 77).

## Conflict of interest

The authors declare no competing interests.
