# Supplementary material for: Targeted Proteomic Quantitation of NRF2 Signaling and Predictive Biomarkers in HNSCC
Source: Mol Cell Proteomics. 2023 Sep 15;22(11):100647. doi: 10.1016/j.mcpro.2023.100647 (PMC10587640; doi:10.1016/j.mcpro.2023.100647)
Supplement: Supplemental Figures [file mmc1.pdf]

SF1

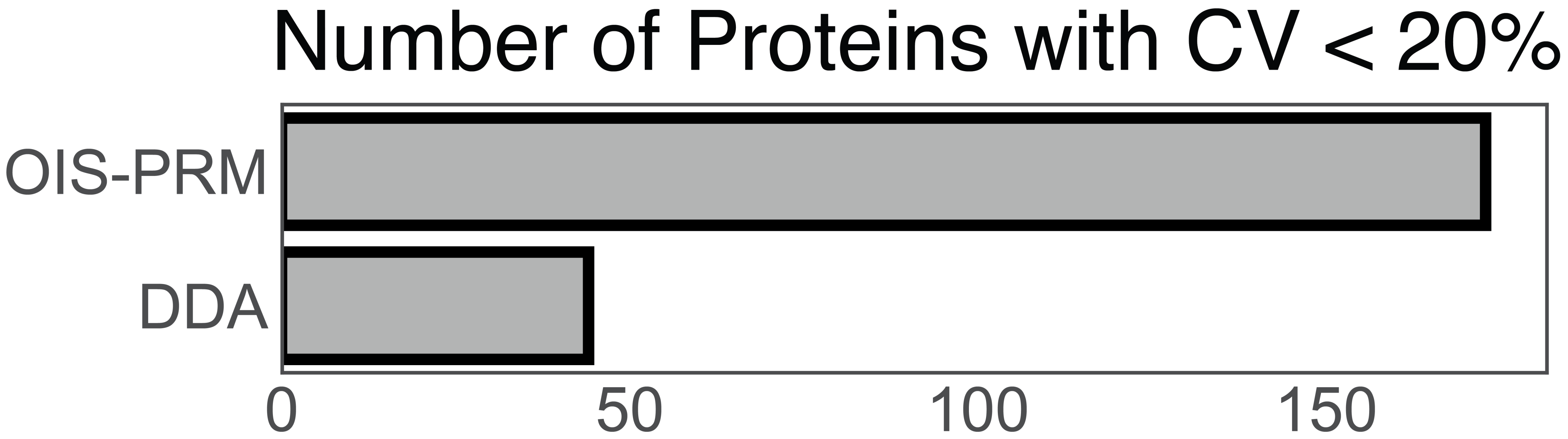

OIS-PRM Improves Sensitivity over DDA. OIS-PRM quantifies more proteins than does DDA (172 vs. 47) a with a coefficient of variation (CV) less than 20%. See main figure 1.

SF2

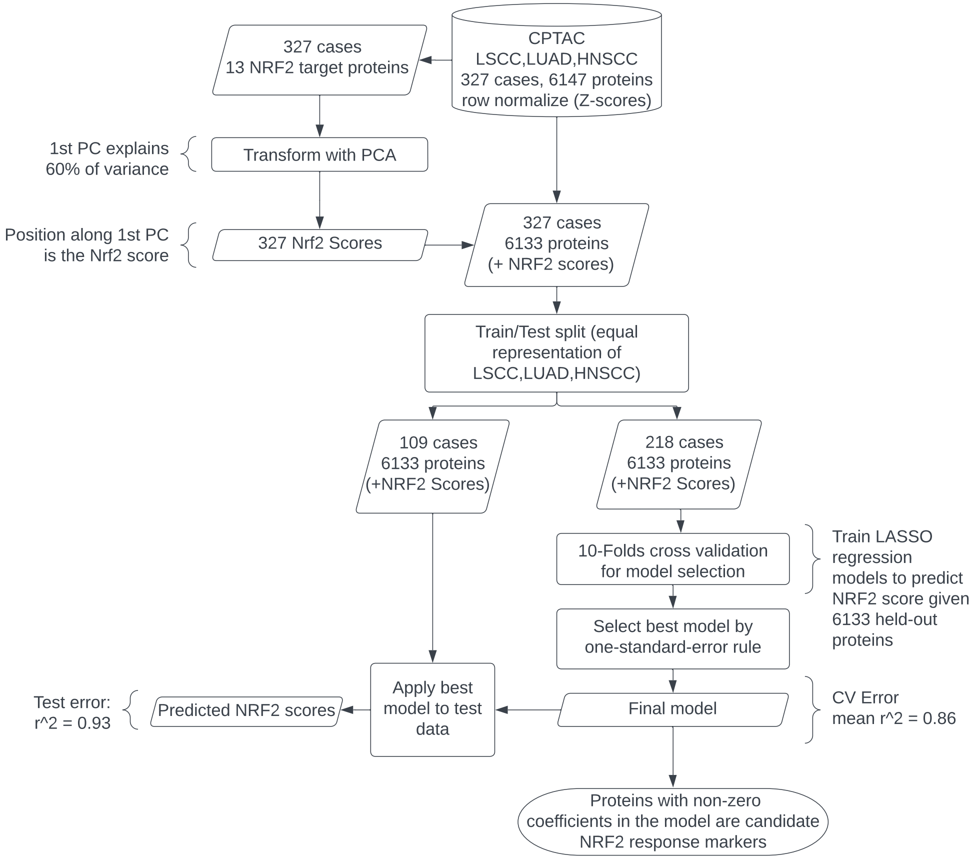

CPTAC Analysis. Schema of NRF2 pathway analysis from CPTAC cohorts for HNSCC, LUSC, and LUAD. See main figure 2.

SF3

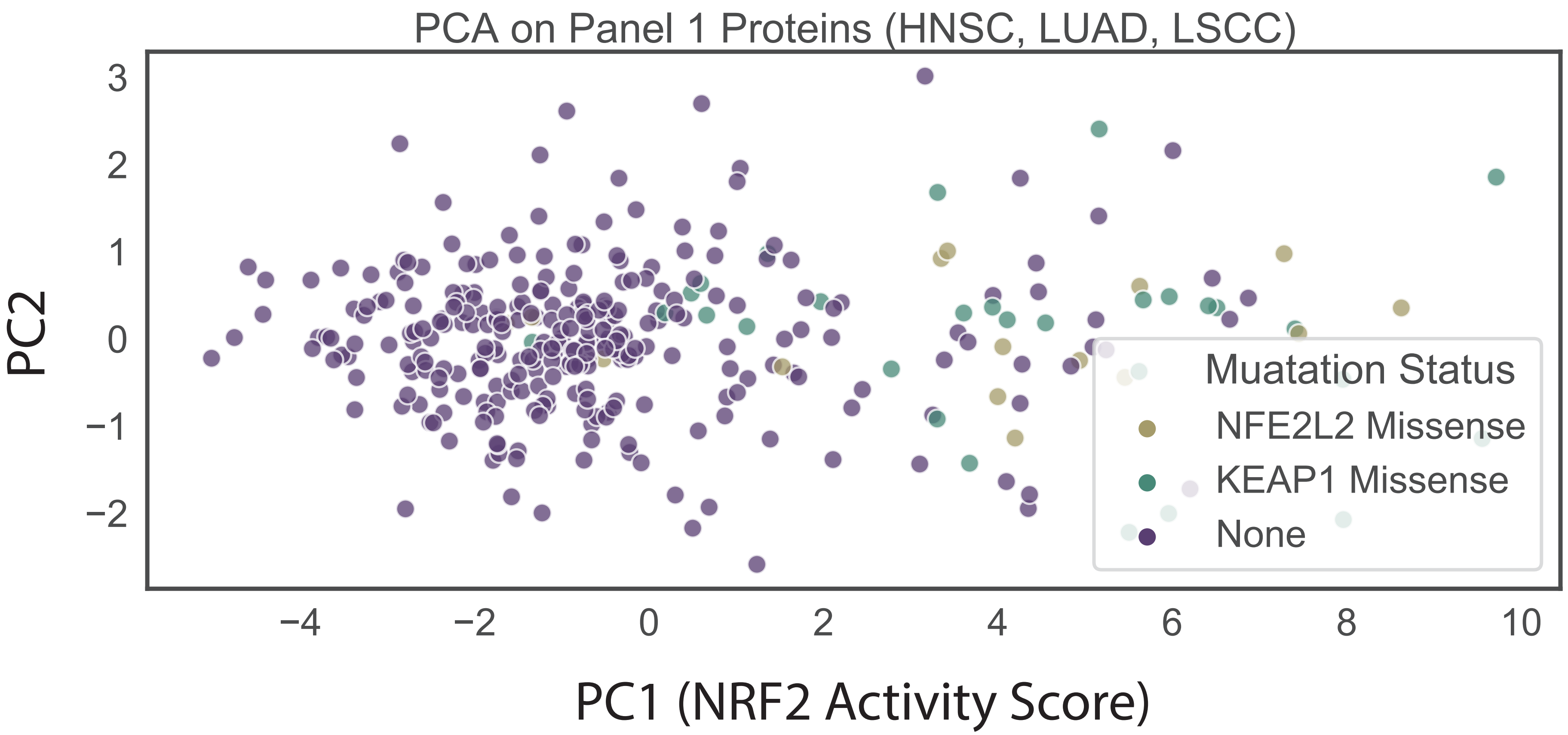

CPTAC NRF2 Targets Expression PCA Plot: A PCA plot of the CPTAC data from figure 2C but demarking mutation status rather than cancer type

SF4

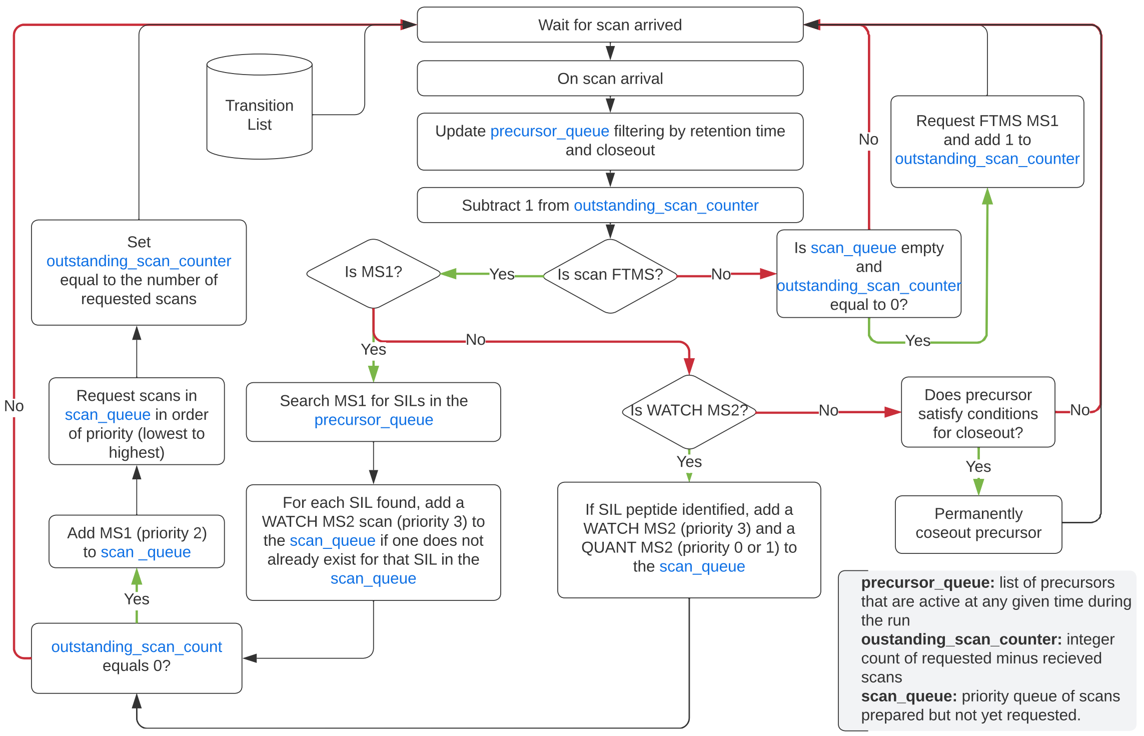

OIS-PRM Algorithm. Schematic of the OIS-PRM data acquisition algorithm as detailed in supplemental methods.

SF5

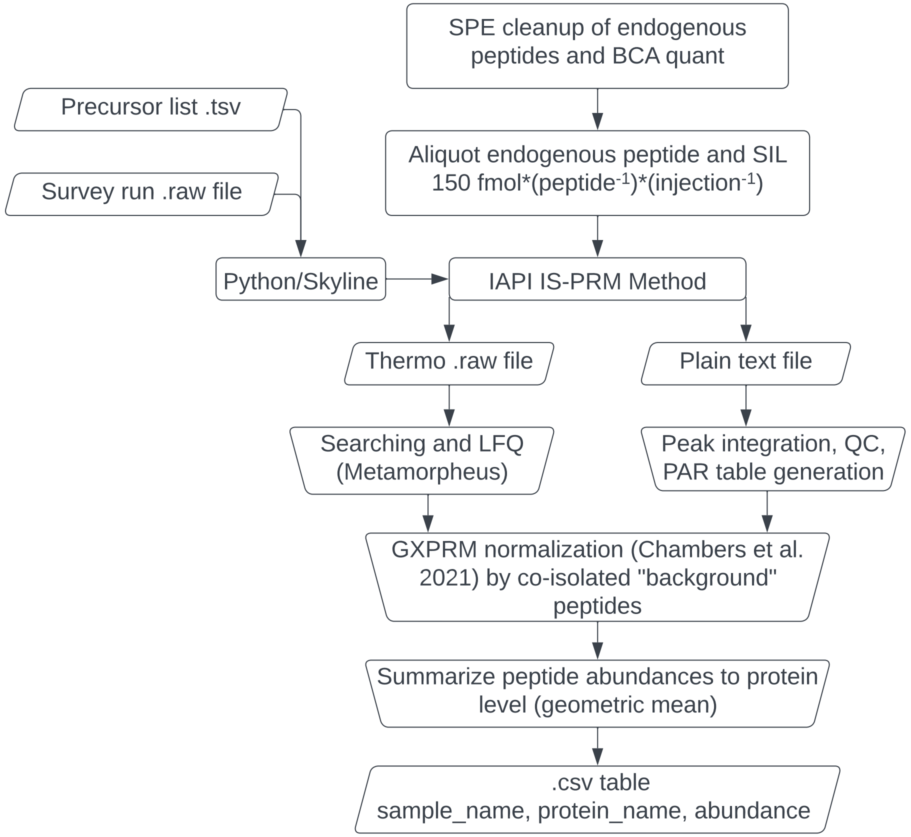

OIS-PRM Analysis Pipeline. Schematic of the data analysis pipeline for OIS-PRM and SureQuant^{TM} experiments as detailed in supplemental methods.

SF6

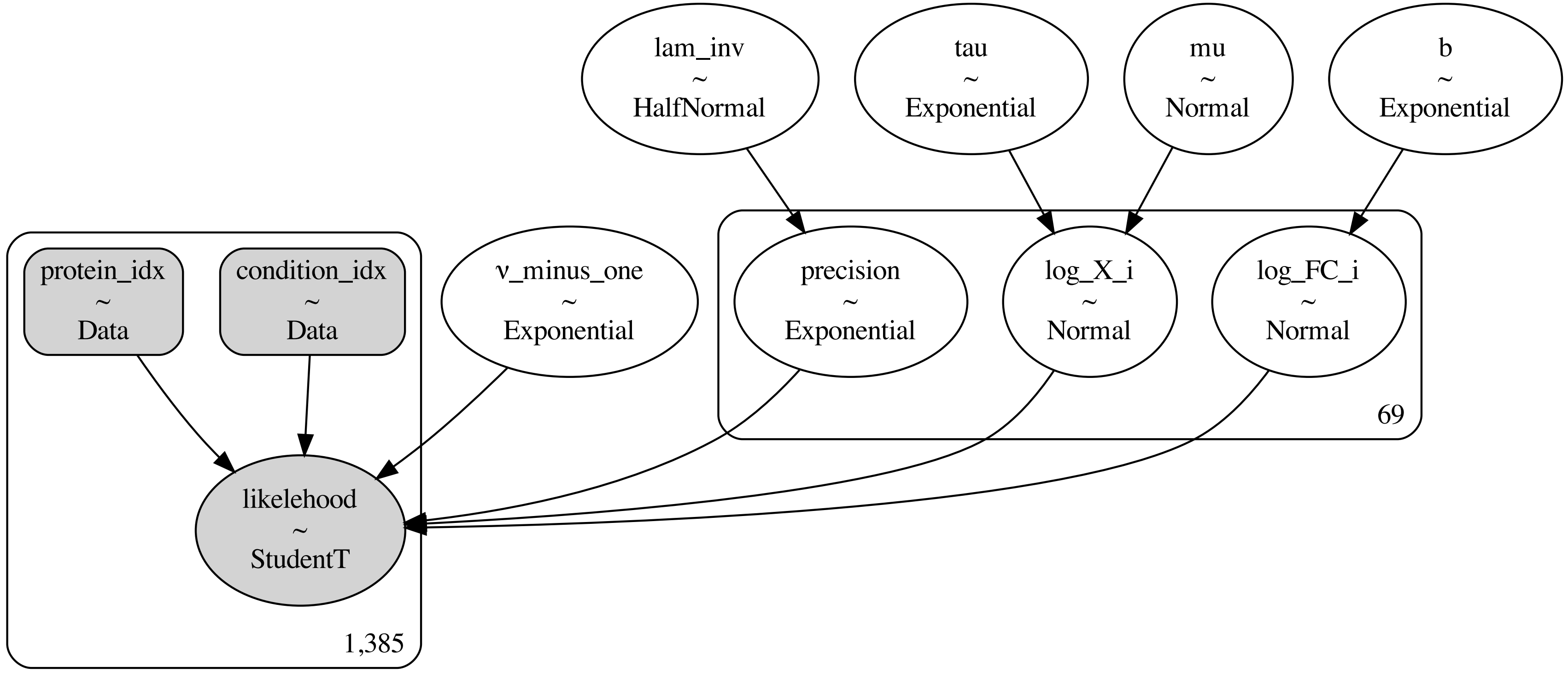

NRF2 Target Expression Model. Plate diagram for hierarchical Bayesian model used to estimate posterior distributions for mean fold changes in the expression of NRF2 targets between NRF2 active and inactive cell lines and tumors. Detailed in supplemental methods.

SF7

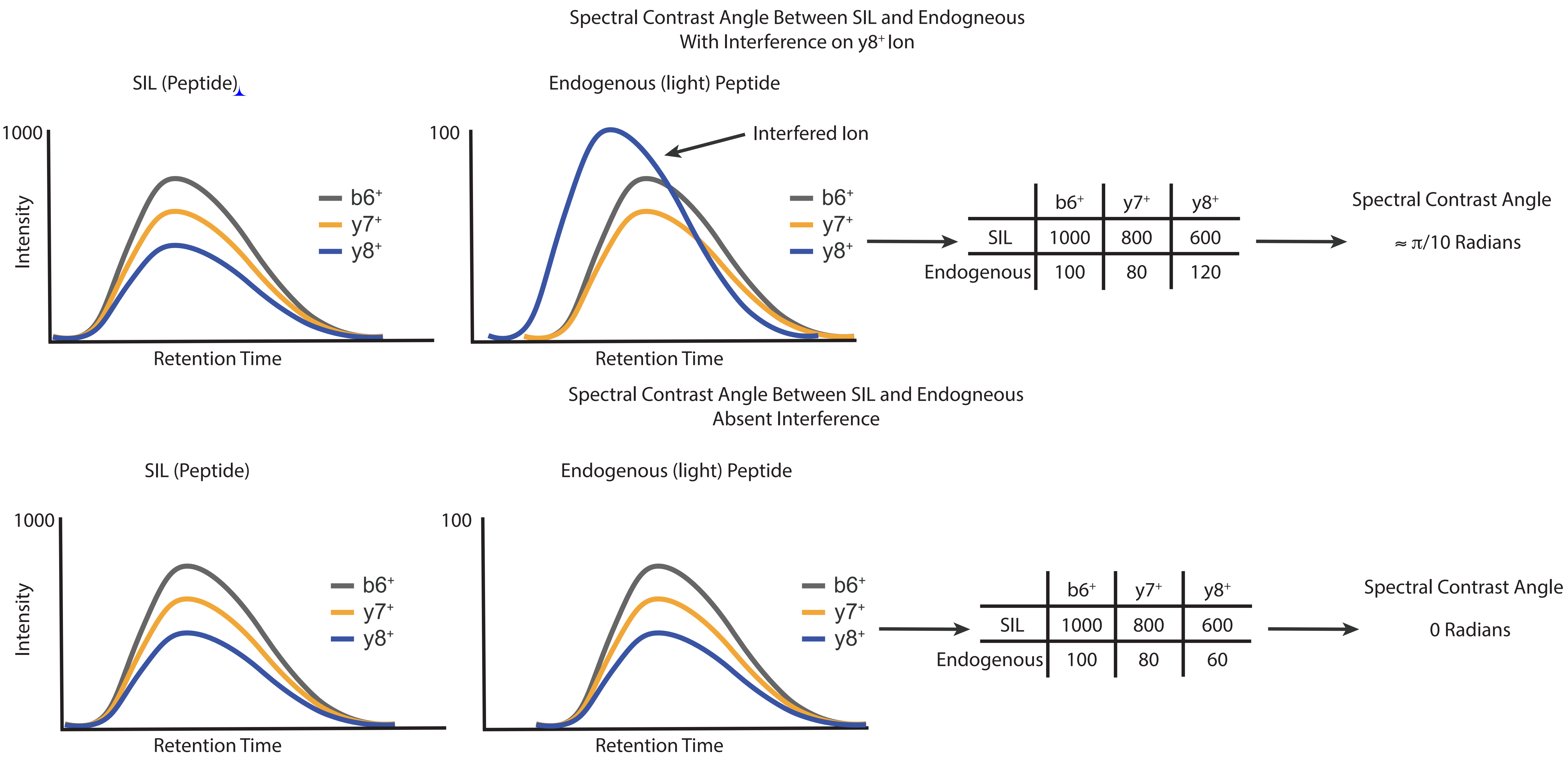

Use of Spectral Contrast Angles to Detect Interference: Spectral contrast angles between the endogenous and internal standard peptides are used to detect interefered transitions.
