# Supplementary material for: Targeted Proteomic Quantitation of NRF2 Signaling and Predictive Biomarkers in HNSCC
Source: Mol Cell Proteomics. 2023 Sep 15;22(11):100647. doi: 10.1016/j.mcpro.2023.100647 (PMC10587640; doi:10.1016/j.mcpro.2023.100647)
Supplement: Table legends [file mmc7.docx]

**Legends for Supplemental Tables**

**Supplemental Table 1. SIL Peptide Catalogue.** Table of SIL peptides included in the HNSCC SIL peptide array. Gene names, Swiss-Prot accession numbers, and peptide sequences are provided. The rightmost column is the peptide specific maximum injection time used in the OIS-PRM method for results reported in this manuscript.

**Supplemental Figure 2. Supporting Evidence for NRF2 Targets.** Catalogue of supporting evidence for many NRF2 associated proteins. Sheet 1 compiles evidence derived from the analysis of CPTAC cohorts as described in the manuscript and supplemental figure 2. Sheet 2 lists the 23 proteins represented by the preliminary SIL peptide array as described in the manuscript. Sheet 3 compiles literature evidence (non-exhaustive) supporting the conclusion that each protein is NRF2 associated or is transcriptionally regulated by NRF2.

**Supplemental Table 3. Transition Ion Catalogue.** Table reporting transitions monitored, precursor charges, fragment ions, and retention times for each targeted SIL and endogenous peptide.

**Supplemental Table 4. Peptide Abundance Tables.** Tables reporting normalized peak area ratios for SIL peptides reported in this study.

**Supplemental Table 5. Protein Abundance Tables.** Tables reporting relative protein abundances for proteins targeted in this study.
