# Supplementary material for: Targeted Proteomic Quantitation of NRF2 Signaling and Predictive Biomarkers in HNSCC
Source: Mol Cell Proteomics. 2023 Sep 15;22(11):100647. doi: 10.1016/j.mcpro.2023.100647 (PMC10587640; doi:10.1016/j.mcpro.2023.100647)
Supplement: Supplemental Methods [file mmc8.docx]

**Supplemental Materials and Methods**

**Statistics**

Throughout, correlations were assessed by Spearman’s rank correlation, and Mann-Whitney U tests were used to compare continuous variables sampled from two populations. We applied hierarchical Bayesian analyses to model the expression of NRF2 target proteins for the 21 cell lines and then for the NRF2 genotyped oral cavity tumors. For proteins i=1,…,N in condition j=0,1 and replicate k=1,…,K:

$y_{ijk}\sim T(\alpha_{i}+\delta_{j}\beta_{i}, \tau_{ij},\upsilon+1$)

where $y_{ijk}$ is the base-2 logarithmic abundance in the k’th replicate of the i’th protein in the j’th condition. For NRF2 inactive cell lines or NRF2 WT tumors, j=0, and otherwise j=1. $T$ stands for the student’s t-distribution parameterized by the mean, precision, and degrees-of-freedom respectively. The priors are as follows. First, the mean logarithmic abundance

$$\alpha_{i}\sim N(\alpha,\tau)$$

of the i’th protein under condition j=0 arises from a normal distribution with mean $\alpha$ and precision $\tau$. Likewise,$\beta_{i}$ parameterizes the logarithmic fold change of condition j=1 over j=0 for the i’th protein and arises from a normal distribution centered at zero with precision b.

$$\beta_{i}\sim N(0,b)$$

$\delta_{j}$ is an indicator variable which equals one for the NRF2 active condition and zero otherwise.

$$\delta_{j}=\left\{ \begin{aligned} 0 if j=0 \\ 1 if j=1 \end{aligned} \right.$$

The precision and degrees of freedom have exponential priors.

$\tau_{ij}\sim Exp(\lambda)$; $\upsilon\sim Exp\left( \frac{1}{29} \right)$

The hyperpriors are as follows:

$$\alpha\sim N\left( \mu,\sigma^{2} \right); \tau\sim Exp\left( 2 \right);b\sim Exp\left( 2 \right);\lambda\sim HalfNormal(1)$$

Hyperpriors $\mu$and$\sigma^{2}$ are empirical; they represent the pooled mean and variance of all logarithmic protein abundances for cell lines where j=0. Sampling from the posterior was carried out by a Hamiltonian Markov chain Monte Carlo algorithm as implemented in the pymc3 python package version 3.11.2 [1]. Sampling occurred without divergences. **Supplemental Figure 6** illustrates the model by a plate diagram.

**CPTAC NRF2 Pathway Analysis**

An initial panel of 13 NRF2 target proteins (**Figure 2a**) were selected based on preference and literature review to develop a pilot SureQuant^TM^ method with a small SIL peptide panel (data not shown). These initial proteins were used to identify other putative targets using the Clinical Proteomics Tumor Analysis Consortium (CPTAC) cohorts for lung adenocarcinoma (n = 110), head and neck squamous cell carcinoma (n = 109), and lung squamous cell carcinoma (n=108) as shown in **Figure 2**. Proteomics data were accessed through the python module cptac [2]. Mutation data were accessed through the genomic data commons data portal [3]. Proteins with any missing values were excluded and each data set was normalized protein-wise by subtracting the mean and dividing by the standard deviation. These data were concatenated to a single table of abundances for 6147 proteins in 327 cases. Using only the 13 proteins from the pilot NRF2 panel, we applied principal components analysis to these data (**Figure 2C**). The coordinate of a case along the first principal component was considered its NRF2 score. Those proteins used in the PCA analysis were then dropped from the data set to leave 6133 proteins for 327 cases. The data was split into a training set of two thirds of the data and a test set of the remaining third such that both sets equally represented each cancer subtype. A LASSO regression model was trained to predict the NRF2 score of a case given the abundance of the 6133 remaining proteins. The LASSO regularization parameter was selected by 10 folds cross validation on the training data. The final model was trained on the entire training set and evaluated on the test set (**Figure 2D**). We used the scikit learn python package for LASSO regression and cross validation [4]. Supplementary figure 2 summarizes the analysis.

**Selection of NRF2 Target Proteins and Peptides**

Differential protein expression in response to genetic perturbation, the CPTAC analysis detailed above, and literature review were used to select putative NRF2 target proteins for inclusion in the IS-PRM assay. Proteins with non-zero coefficients in the LASSO regression model to predict the NRF2 score (**Figure 1D-E**) were candidates for selection. These were further reduced to 17 proteins for which the coefficients were largest or for which some literature evidence supported a connection to NRF2 (**Supplemental Table 1**). Differential protein expression between SCC90, SCC152, and SCC154 cell lines stably expressing NRF2 E79Q and their parental lines were assessed by label-free quantification from data-dependent acquisition experiments. Evidence from these three sources were used to select a final protein panel provided in the supplemental data. For each selected protein, three or more peptide representatives were chosen from the ProteomeTools database [5]. When possible, we selected peptides between 7 and 16 amino acids in length and in order of decreasing priority, those not containing methionine, cysteine, or known phosphosites in the PhosphoSitePlus database [6].

**Mixture of 218 SIL Peptides Targeting Head and Neck Cancer**

Stable isotopically labeled (SIL) peptides were obtained in array-purity from Vivitide. In sum, 288 SIL peptides were synthesized with ^15^N and ^13^C-labeled lysine and arginine residues. Assuming for each the maximum manufacturer estimated yield of 0.7 µmol per peptide, we prepared a mixture of all 288 peptides at a nominal concentration of 300 nM/uL per-peptide. We excluded a handful of peptides for which the MALDI spectrum supplied by the manufacturer gave evidence of excessive by-products of synthesis or where the expected singly charged m/z was absent. After initial survey runs, the concentrations of some low-intensity peptides were increased in the pooled mixture. In a final survey run, a total of 218 peptides were observed with a minimum of 5 quantifiable transitions. The final mixture of these peptides was divided into 100 µL aliquots, dried by vacuum centrifugation, and stored at -80°C.

1. Salvatier, J., T.V. Wiecki, and C. Fonnesbeck, *Probabilistic programming in Python using PyMC3.* PeerJ Computer Science, 2016. **2**.

2. Lindgren, C.M., et al., *Simplified and Unified Access to Cancer Proteogenomic Data.* Journal of Proteome Research, 2021. **20**(4): p. 1902-1910.

3. Grossman, R.L., et al., *Toward a Shared Vision for Cancer Genomic Data.* New England Journal of Medicine, 2016. **375**(12): p. 1109-1112.

4. Fabian Pedregosa, G.V., Alexandre Gramfort, Vincent Michel, Bertrand Thirion, Olivier Grisel, Mathieu Blondel, Peter Prettenhofer, Ron Weiss, Vincent Dubourg, Jake Vanderplas, Alexandre Passos, David Cournapeau, Matthieu Brucher, Matthieu Perrot, Édouard Duchesnay;, *Scikit-learn: Machine Learning in Python.* Journal of Machine Learning Research, 2011. **12**: p. 2825-2830.

5. Zolg, D.P., et al., *Building ProteomeTools based on a complete synthetic human proteome.* Nat Methods, 2017. **14**(3): p. 259-262.

6. Hornbeck, P.V., et al., *PhosphoSitePlus, 2014: mutations, PTMs and recalibrations.* Nucleic Acids Res, 2015. **43**(Database issue): p. D512-20.
